# Predator-induced renesting and reproductive effort in indigo buntings: more work for less pay?

**DOI:** 10.1093/conphys/cou063

**Published:** 2015-02-05

**Authors:** Dana L Morris, John Faaborg, Brian E Washburn, Joshua J Millspaugh

**Affiliations:** 1 Division of Biological Sciences, University of Missouri, 110 Tucker Hall, Columbia, MO 65211, USA; 2 Division of Science, Mathematics and Computer Science, Central Methodist University, 411 Central Methodist Square, Fayette, MO 65248, USA; 3 Department of Fisheries and Wildlife Sciences, University of Missouri, 302 Anheuser-Busch Natural Resources Building, Columbia, MO 65211, USA; 4 United States Department of Agriculture, Wildlife Services, National Wildlife Research Center, 6100 Columbus Avenue, Sandusky, OH 44870, USA

**Keywords:** Avian breeding biology, corticosterone, forest fragmentation, nest success, post-breeding, renesting

## Abstract

Renesting after nest predation is ultimately an adaptive response to increase productivity in birds. However, renesting also increases reproductive effort to replace lost clutches. We investigated the consequences of this increased reproductive effort by determining whether renesting in female indigo buntings (*Passerina cyanea*) is associated with a decline in body condition (size-corrected mass) and haematocrit and an increase in stress hormones and whether renesting or maternal body condition is associated with a decline in productivity (clutch size, nestling body condition). Next, because a consequence of multiple renesting attempts is a prolonged breeding season and later timing, we predicted that a population of post-breeding females and juveniles would have lower body condition in fragmented forest than in contiguous forest owing to higher nest predation and frequency of renesting. Both forest types were settled by females of similar condition. Nest survival was lower in fragmented forest, where a higher proportion of females failed their first attempt and the breeding season was 2 weeks longer. Compared with females on their first attempt, renesting females had lower body condition and haematocrit and higher corticosterone concentrations. Lower maternal body condition was associated with higher concentrations of corticosterone, lower nestling body condition and smaller clutches. Clutch size was lower in renests and in fragmented forest. Nestling condition was lower in renests but did not vary greatly with forest type. Despite a prolonged breeding season in the fragmented forest, post-breeding females and hatch-year birds were in similar condition in both forest types. Our results suggest that the indirect effects of nest predation on maternal and offspring condition pose additional individual-level costs that have not been considered in the context of fragmentation studies. We discuss how predator-induced renesting could have additional demographic consequences by prolonging the breeding season and prompting seasonal interactions or carry-over effects that could impact populations.

## Teaser

Renesting after nest predation is an adaptive response to increase productivity in birds. However, renesting female Indigo Buntings had lower body condition, higher stress hormone concentrations, and lower productivity than females successful on their first attempt. The frequency of renesting was higher in fragmented forest than in contiguous forest.

## Introduction

Nest predation accounts for most reproductive failure in birds (Martin, 1995), and many studies have linked reduced reproductive success to population-level consequences (Robinson *et al*., 1995; Lloyd *et al*., 2005). Such predation is likely to impact current fitness and has the potential to create additional impacts on individuals because nest loss in many species invokes renesting. The importance of renesting to measures of seasonal fecundity has been demonstrated (Nagy and Holmes, 2004; Gryzybowski and Pease, 2005), as have the effects of renesting on return rates and decision rules of breeding adults (Haas, 1998; Hoover, 2003; Schmidt and Whelan, 2010). With the exception of some recent studies of renesting on maternal performance (Nager *et al*., 2001; Travers *et al*., 2010), relatively little attention has been directed to the physiological constraints on breeding females imposed by renesting. Compared with females that raise a brood on their first attempt, females that experience predator-induced renesting necessarily experience greater reproductive effort by working more to produce young and breeding for longer due to later nest initiation dates with each successive attempt.

A possible consequence of renesting is a change in circulating concentrations of corticosterone. The secretion of corticosterone is an adaptive process that allows organisms to respond quickly to unpredictable stressors (Romero, 2002; Breuner *et al*., 2008; Bonier *et al*., 2009) and increases the potential for survival by stimulating gluconeogenesis (Sapolsky *et al*., 2000). Although there is much variation in the response of chronically stressed wild animals (Dickens and Romero, 2013), high baseline or stress-induced concentrations of corticosterone have been linked to low food availability (Kitaysky *et al*., 1999), low body mass and habitat quality (Marra and Holberton, 1998), intense silviculture (Leshyk *et al*., 2012, 2013), reproductive effort (Bonier *et al*., 2011), experimental increases in nest predation (Travers *et al*., 2010) and perceived predation risk (Scheuerlein *et al*., 2001; Clinchy *et al*., 2004; Fontaine *et al*., 2011) and can be related to low fitness (Bonier *et al*., 2009). Abnormally low baseline or stress-induced glucocorticoid concentrations could indicate that individuals are unable to respond to energetic needs (Busch and Hayward, 2009).

The frequency of renesting could be especially high for populations of songbirds nesting in fragmented habitat that attracts nest predators (Dijak and Thompson, 2000; Chalfoun *et al*., 2002). Life-history theory suggests that high rates of nest predation select for smaller clutches to conserve energy and maximize the likelihood of renesting after nest failure (Slagsvold, 1984) or to balance risk and investment with each nesting attempt (Skutch, 1949; Martin, 1995). More recent empirical work has demonstrated the physiological constraints behind these behavioural trade-offs (Williams, 2005; Love and Williams, 2008). Egg production increases resting metabolic rate (Vézina and Williams, 2005), blood parasitaemia (Knowles *et al*., 2009) and reproductive anaemia (Williams *et al*., 2004; Wagner *et al*., 2008) in breeding females. Experimentally elevated egg production negatively affects maternal condition (Monaghan and Nager, 1997; Sanz, 1999; Travers *et al*., 2010), the capacity to rear chicks (Monaghan *et al*., 1998) and subsequent clutch size (Travers *et al*., 2010) and demonstrates that increased reproductive demand causes an increase in reproductive effort and baseline corticosterone (Bonier *et al*. 2011).

Consequently, a prolonged breeding period, which could be driven by renesting, could indirectly impact adults and juveniles owing to declining habitat suitability (Nagy and Holmes, 2005), later onset of moult and migration (Done *et al*., 2011; Stutchbury *et al*., 2011) and lower offspring survival and recruitment (Verboven and Visser, 1998). For migratory birds, these potential costs and delays could trigger changes in energetic condition that impact subsequent life-history stages (Nager *et al*., 2001; Visser and Lessells, 2001; although see Haas, 1998 and Stutchbury *et al*., 2011). Such costs at the individual level could also cascade into population-level consequences if interseasonal survival or future reproduction subsequently decline through carry-over effects from one life-history stage to the next (Norris *et al*., 2004).

Here, we model the prediction that renesting is related to a decline in maternal condition and productivity. Previous work in Missouri shows that the indigo bunting (*Passerina cyanea*) has low nest success (15–20%) in fragmented forests (Dearborn, 1999; Burhans *et al*., 2002) and higher success (43%) in contiguous forests (Morris *et al*., 2013). Working in these same forests, we measured nest survival and a suite of maternal parameters, including size-corrected mass (body condition), haematocrit, baseline corticosterone and acute corticosterone. Given the highly dynamic role of glucocorticoids in the stress response (Dickens and Romero, 2013) and that baseline glucocorticoids can shift with life-history stage and energetic state (Bonier *et al*., 2009), we predicted that the higher energetic demand of renesting is associated with higher baseline corticosterone and short-term increases in stress-induced corticosterone. We also modelled the prediction that renesting and reduced maternal condition result in lower productivity (clutch size and nestling body condition). We expected a greater frequency of renesting and a longer breeding season in the fragmented forests due to lower nest survival. To examine the effects of a longer breeding season on females and later timing on juveniles, we measured body condition of a population of post-breeding females and hatch-year birds in both forest types. We discuss the implications of our findings in relationship to individual-level and demographic consequences of nest predation.

## Materials and methods

### Study sites

We studied indigo buntings, hereafter buntings, in a contiguously forested landscape and a fragmented landscape, separated by ∼320 km. Indigo bunting females solely choose nest sites, construct nests, incubate and provision nestlings, although male buntings typically help to provision fledglings (Payne, 2006; D.L.M., personal observation). Both landscapes are 200–300 m above sea level. We sampled in the contiguous forest in the Ozarks region of southeast Missouri, USA during three breeding seasons (2000–2003) at three sites (each ∼3000 ha) in the Current River Conservation Area (11 331 ha; 37° 19′ N, 91° 00′ W; 275 m elevation), a 40 000 ha contiguous oak-hickory forest. We studied buntings during one breeding season (2003) at two sites in fragmented forests, Davisdale (1125 ha; 39° 00′ N, 92° 62′ W; 180 m elevation) and Rudolf Bennitt (1460 ha; 39° 25′ N, 92° 45′ W) Conservation Areas, where each site consisted of ∼30% forest cover with 400 ha of forest surrounded by grazing pasture, old field, hayfield and row crop agriculture. Within forest types, sites were >5 km apart to maintain independent observations. To prevent possible microclimatic edge effects, such as soil or ambient air temperatures or light levels (Chen *et al*., 1999), from confounding with forest type, we monitored breeding buntings in early successional habitat within the forest interior at all sites, including silviculture treatments (3–13 ha clearcuts or 21- to 43-m-diameter group-selection cuts, all 4–6 years old), forest-interior roadsides (20–25 m wide) and wildlife food plots. Food plots are small forest openings (0.25–2 ha) manipulated by mowing, disking and planting to promote growth of grass and vegetative food resources for wildlife. In the contiguous forest, our clearcut and group-selection plots were located within the study sites of the Missouri Ozark Forest Ecosystem Project, an experimental study of forestry techniques on forest flora and fauna (Brookshire and Shifley, 1997). In the fragmented forest, group-selection plots were located within firewood removal sites. The fragmented forests were located 280 km north of the large contiguous Ozark forest, resulting in a phenological difference where nest initiation began 2 weeks later in the fragments.

### Reproductive effort

Nests were located using behavioural cues (Martin and Geupel, 1993) and systematic searches of known territories. We recorded the location and boundaries of bunting territories on topographical maps. We monitored singing males, noted the presence of females and/or fledglings and searched for nests every 1–3 days from late April to August. A single territory (<2 ha) existed in most wildlife food plots and group-selection openings, whereas we found a linear succession of territories along roads and two to six territories in each clearcut. Given that we visited territories every 1–3 days, mapped territory boundaries and matched new nests in a territory according to nest stage and days since last failure, we were confident that we monitored individual nesting attempts for 80 females, representing about 20 females per breeding season. For all other nests, we categorized nesting attempt into first nests and renests based on earliest nest initiation dates and the time to renest after predation in each forest type. Nests initiated after 19 May in the unfragmented forest or 9 June in the fragmented forest were considered renests. Females typically spend 2–8 days building nests, 3–4 days laying, 12 days incubating and ∼10 days feeding nestlings before fledging (Payne, 2006; D.L.M., personal obseration). Females typically renested within 3 days of losing a clutch or brood. With a crew of two or three field assistants, we spent 700 h searching for and monitoring nests each season. Working on the Missouri Ozark Forest Ecosystem Project study sites increased access to additional nests located and monitored by their crew of 15 interns.

Given that our measures of productivity between forest types did not overlap each year, we were concerned that annual variation in rainfall or air temperature could have contributed to our findings. However, monthly air temperature and rainfall between forest types did not vary between 2003 in the fragmented forest (maximal temperature = 26.6°C; minimal temperature = 14.6°C; rainfall = 9.65 cm) and 2000–2003 in the contiguous forest (maximal temperature range = 27.5–28.3°C; minimal temperature range = 16.3–17.2°C; rainfall = 8.4–12.9 cm).

### Pre-breeding condition

Before comparing breeding condition between the two forest types, we first determined whether the fragmented and contiguous forests were settled by a similar proportion of older and younger females and whether body condition varied at the time of arrival. Before the initiation of nesting in each forest, we used five 12 m mist nets placed end to end, to capture a subset of pre-breeding females (i.e. showing incomplete brood patches) between 06.00 and 10.00 h in wildlife food plots or fields within the study areas where they congregated to feed in April or early May. We aged females (SY = first breeding season, ASY = second/subsequent breeding season) by plumage characteristics according to the method of Pyle (1997), measured (unflattened wing chord and tarsus length, to 0.1 mm), weighed (nearest 0.5 g) and individually marked them with coloured plastic and numbered US Fish & Wildlife Service bands.

### Breeding condition

During the breeding period, we captured females at nests, at which time we measured and individually marked them and collected blood samples. We did this on nestling day 6 based on our preliminary findings that 75% (3 out of 4) of females abandoned their nests if handled and bled prior to nestling day 6. We also chose the middle of the nestling period because it represents a period of maximal investment and energetic costs by female buntings (Payne, 2006). In cases of nest depredation prior to day 6, we monitored subsequent attempts, as described above, and gathered physiological data once a pair raised a brood to the age of 6 days. To minimize the effects of disturbing the female prior to capture, we placed 6 m mist nets (closed) within 1–3 m of the nest 1 day prior to capture so that the parents could acclimate to the presence of the nets. Presumably, any corticosterone secretion elicited by this disturbance would be physiologically cleared by the following day (Wingfield *et al*., 1992). Between 06.00 and 10.00 h on the morning of capture, we waited until females left their nest on a foraging trip, then opened the net and passively captured them when they returned to the nest. Within 3–5 min of entering a net, we used a 26-gauge needle to puncture the female's brachial vein and collect 50 µl of blood into heparinized microcapillary tubes. We used this sample as a measure of baseline corticosterone. Females were held in cloth bags, and a subsequent sample collected at 30–35 min post-capture provided a profile of the acute stress response to capture and handling (Wingfield *et al*., 1992). We measured and banded females and nestlings (as described above) in between blood samples. Blood samples were kept on ice until centrifuged in a microhaematocrit centrifuge (10 000 *g*, 10 min) within 2–5 h of collection. Plasma was drawn off using a Hamilton syringe and stored at −20°C until assayed. We measured haematocrit (packed red blood cells/total volume) on each baseline sample using callipers (to the nearest 0.01 mm) before collecting plasma. In the case of multiple tubes per sample, we recorded the average haematocrit.

### Post-breeding condition

To examine the effects of a longer breeding season, we measured body condition of a subset of post-breeding females and hatch-year birds by capturing post-breeding adults and hatch-year (juvenile) birds in mist nets as they began abandoning territories and congregating in wildlife food plots within the study sites. Given that we trapped birds according to biological post-breeding time lines, we are fairly confident that we were not measuring migrants. For example, in each forest, after carefully monitoring territories, finding no new nests and hearing few singing males, we set up five or six 12 m mist nets in adjacent fields and food plots. This occurred in late July (2002 and 2003) in the unfragmented sites and in mid-August (2003) in the fragmented sites. We identified post-breeding birds by signs of moult and/or recession of brood patches and cloacal protuberances and measured birds as described in Pre-breeding section above. Wildlife food plots were located within 100 m from breeding sites in the fragmented forest and 1–5 km from breeding sites in the unfragmented forest, resulting in few recaptures between breeding stages.

### Hormone assays

We assayed plasma samples for total corticosterone concentrations in duplicate using a commercially available I^125^ radioimmunoassay (RIA) kit (MP Biomedicals, Costa Mesa, CA, USA). We followed the manufacturer's method, except that we halved the volume of all reagents and diluted samples 1:50 with steroid dilutent prior to assay. Parallelism and recovery of exogenous corticosterone validation assays were conducted to validate the utility, accuracy and precision of this particular radioimmunoassay kit for use with a suite of passerine birds, including buntings (Washburn *et al*., 2002). We ran separate assays in each year of the study (2000–2003). Intra-assay variation was calculated using five known concentration standards from the kit; yearly intra-assay coefficients of variability for quality controls were 12.5, 3.7, 8.9 and 15%, respectively. Additionally, we ran two to four controls from the radioimmunoassay kit in each assay and had an interassay variation across all years and assays of 6.9%. Intra-assay variation calculated from 60 randomly chosen samples across all assays was 3.9%.

### Statistical analyses

We used the logistic exposure method (Schaffer, 2004) to model nest survival as a function of forest type. To examine our prediction that birds in the fragmented forests are more likely to fail on their first attempt, we estimated nest survival between forest types using first nest attempts only. The sampling unit with this method is the interval between nest checks. The effective sample size for the model is derived from the model likelihood and consisted of the sum of the number of days that all nests were known to have survived and the number of intervals that ended in failure (Rotella *et al*., 2004). We were interested in the main effect of forest type on nest survival, but other temporal effects, such as nest stage and date, can strongly influence nest survival (Cox *et al*., 2012). To account for these possible nuisance parameters, we first evaluated a set of three single-variable models including nest stage (incubation or nestling), day of year and habitat type (silviculture, road, food plot) to see which best fitted the data (Supplemental Table S1). Maternal body condition could also influence nest survival, so we evaluated it as a possible nuisance parameter (Supplemental Table S1). Given that maternal body condition was measured only during the nestling stage, we compared this single-variable model to a constant survival model separately from the other nuisance parameters. We then used the variable from the top-ranked model(s) as a covariate in the forest model and compared it with a constant survival model. We used the second-order Akaike information criterion (AIC_c_; Burnham and Anderson, 2002) for model selection and calculated differences in AIC_c_ values and Akaike weights to evaluate the relative support for each model. The model selection approach ranks models according to the amount of information explained while accounting for model complexity, therefore allowing robust comparisons of competing hypotheses. Models with low AIC_c_ scores, ΔAIC_c_ < 4, and high Akaike weights represent best-fit models. To account for model selection uncertainty (Burnham and Anderson, 2002), we report 95% confidence intervals (CIs) for parameter estimates based on model-averaging over the candidate model set. We calculated nest survival probability over the entire nest period in terms of the effects, by raising daily survival to a power equal to the average length of the nest cycle (26 days for buntings).

We calculated an estimate of the proportion of females that renested based on the sample of females that had known nest attempts. We compare differences using 95% CIs, which can be more informative than *P*-values, particularly when parameter estimates and their errors are of interest (Johnson, 1999). We calculated earliest and latest nest initiation dates to determine breeding season length in both forested areas.

We calculated separate body condition indices as the residual mass from an ordinary least-squares regression relating body mass to tarsus length for females, nestlings and juveniles (Schulte-Hostedde *et al*., 2005). Body condition was expressed as the size-corrected body mass (in grams). Individuals with residuals > 0 were considered heavy given their size, and those with residuals < 0 were lean.

We used the same second-order Akaike information criterion for model selection described above to evaluate four maternal variables [mass-corrected size (hereafter maternal body condition), haematocrit, baseline corticosterone and acute corticosterone] and two productivity variables (clutch size and nestling condition) as a function of nesting attempt (i.e*.* first or renest, if observed renesting one or more times after nest failure) and forest type (contiguous or fragment). We evaluated maternal and productivity variables using a full data set where nesting attempts were categorized as first or renest and a subset of data with known nesting attempts. To retain parsimony in the final models with attempt and forest type, we first ran a set of six single-variable models including maternal age, day of year, nestling age, brood size, habitat and brood parasitism (yes/no). To control for variation in elapsed time of blood collection, for corticosterone, we added the parameters Time1 (elapsed time from capture to baseline blood collection) and Time2 (elapsed time from capture to acute corticosterone blood collection). Parameters with ΔAIC_c_ < 4 were included as nuisance parameters in every model of the candidate set (Supplemental Tables S2 and S3). Baseline and acute corticosterone concentrations were logarithmically transformed to correct for non-normality, although we present parameter estimates as the inverse natural logarithm to ease interpretation. We ran separate preliminary general linear models with site and year alone with each response variable. There were no differences among sites within forest types or among years within the contiguous forest (*P* > 0.05 for each response variable). Sample sizes vary for each measure of condition, largely due to the difficulty of collecting sufficient blood samples.

For each response variable, we generated five to nine additive and multiplicative general linear or general linear mixed models. For maternal condition, we included a global model (forest, attempt and forest × attempt interaction), a full model (forest and attempt without interaction), a forest-only model, an attempt-only model, and an intercept-only model. In the context of energy constraints, we investigated whether corticosterone or productivity varied with endogenous stores of energy (Wingfield, 1994; Sockman and Schwabl, 2001; DuRant *et al*., 2013) by including body condition index as a covariate and the interaction nesting attempt × maternal body condition in the competing models for maternal corticosterone concentrations, nestling body condition and clutch size. For nestling condition, we used a general linear mixed model with individual nestling as the sampling unit and nest ID as a random effect.

Increased rates of brood parasitism associated with forest fragmentation (Robinson *et al*., 1995) may also increase the energetic demands of breeding females (Dearborn *et al*., 1998). Our estimates of brood parasitism were low in comparison to other estimates from the Midwest (Suarez *et al*., 1997; Peak *et al*., 2004), with 11% (44 of 407) and 21.1% (22 of 104) of nests parasitized in the contiguous and fragmented forests, respectively. Small sample sizes of females measured at parasitized nests (*n* = 9) prevented us from including parasitism as a parameter in the models and thus, we based all models on unparasitized nests only.

Three of 80 females attempted to raise a second brood after successfully raising a brood on their first attempt, and for these, we included data from their first brood only. Breeding site fidelity was low (15 of 154 colour-banded females returned to territories), but to maintain independence, we used data from their first observed year.

To examine differences in body condition among pre-breeding, breeding and post-breeding females, we used a model selection approach as described above. As we were particularly interested in the interaction between forest type and period (pre-breeding, breeding and post-breeding), we specified only two models; a null model and a global model with forest type, period and forest × period interaction. Very few individuals were repeatedly sampled between periods (13 females, of which three were captured in each period), precluding us from using a repeated-measures approach, so we used a female's first occurrence and considered birds in each period as independent samples. To examine differences in juvenile body condition between forest types during the post-breeding period, we used a similar model selection approach, using a null model and a global model containing forest type only.

We present parameter estimates (β) with 95% CIs from the best model or, in cases of model selection uncertainty, model-averaged estimates and unconditional 95% CIs from a confidence set of models where combined Akaike weights were ≥0.95 (Burnham and Anderson, 2002). In cases of model selection uncertainty, we calculated relative variable importance by summing the Akaike weights across all models in the set where a variable occurred. Larger weights indicate more importance of that variable, relative to other variables (Burnham and Anderson, 2002). Models in tables are ranked in ascending order by ΔAIC_c_ value, which is the difference in AIC_c_ score between the best model and subsequent models. The number of parameters (*k*), and Akaike weights (*ω*) are given for each model. We performed all analyses with SAS, version 9.3 (SAS Institute, 2010).

## Results

### Reproductive effort

We found 407 bunting nests in the contiguous forest and 104 in the fragmented forest. Our nest survival estimate from first attempts is based on 1784 observation days from 191 nests in the contiguous forest and 38 nests in the fragmented forest. We found no evidence that variation in nest survival was related to maternal body condition or habitat (Supplemental Table S1), but stage and day were included in the final model with forest type. The forest model was higher ranked than the constant survival model (Table 1). Period survival was 40% (95% CI, 28–51%) in contiguous forest and 21% (95% CI, 8–39%) in fragmented forest. We followed individual nesting attempts for 80 females. Of these, the proportion of renesting females in the forest fragments was 73% (16 of 22) and in contiguous forest 12% (7 of 58). Despite the phenological difference between the two forest types, the breeding season was 2 weeks longer in the fragmented forest based on earliest (29 April in contiguous vs. 16 May in fragmented forest) and latest nest initiation dates (13 July in contiguous vs. 10 August in fragmented forest). Although buntings renest up to 1 km from their original territories (Payne, 2006), we did not detect new females or territorial males moving into our study sites later in the season. Given the large geographical separation between the two forest types, it is unlikely that forest birds were moving to fragments. We found no evidence that females delay their first nesting attempt after arrival in either forest type, based on regular monitoring and mapping of territories.

**Table 1:** Model selection results of logistic exposure analysis of survival of indigo bunting nests in Missouri, 2000–2003

Model	*k*	ΔAIC_c_	*ω*
Forest type + stage + day	5	0.0	1.00
Constant survival	2	18.3	0.00

### Maternal condition

**Figure 1:**
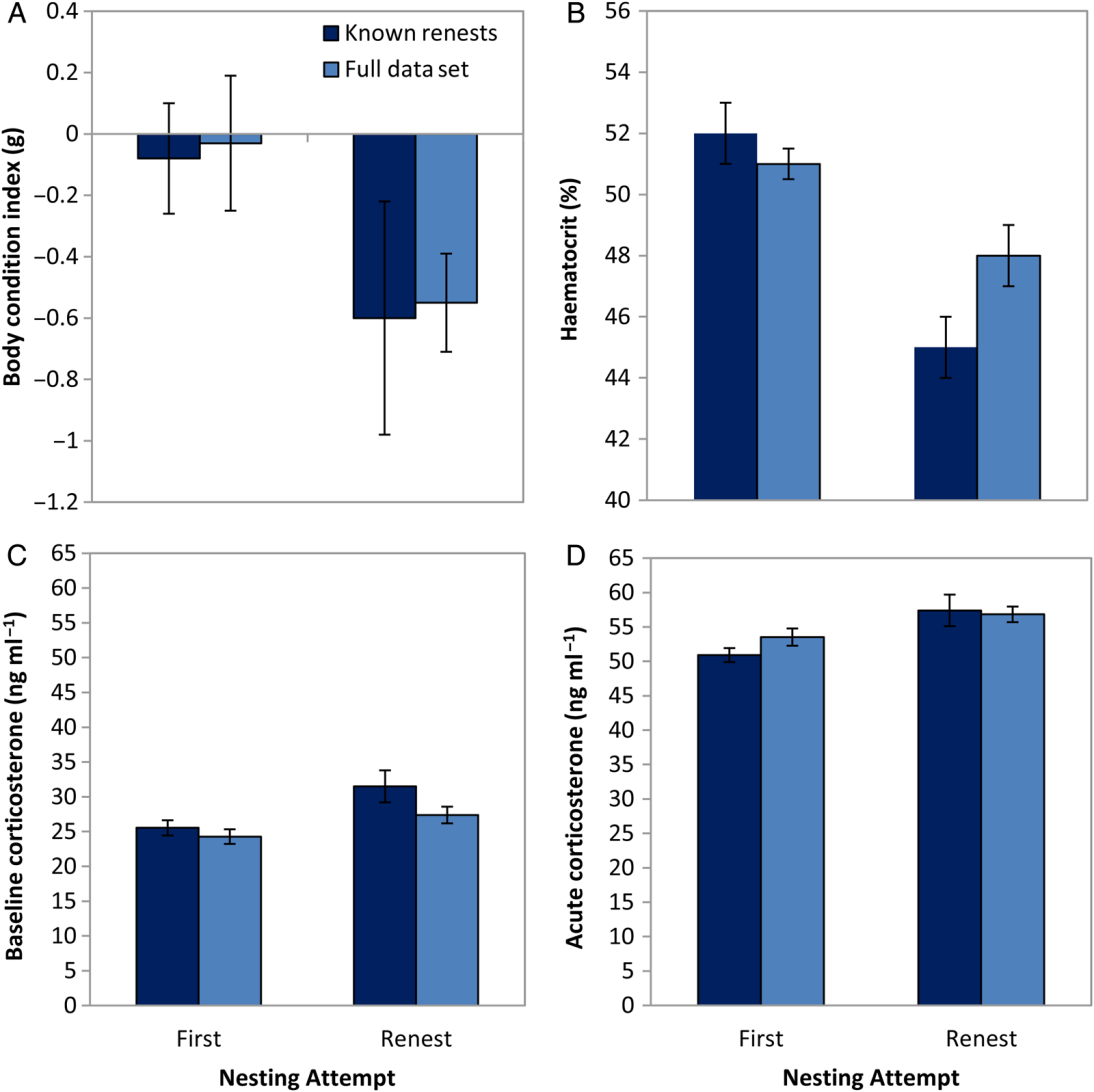
Physiological measures of female indigo buntings with nesting attempt. Parameter estimates [β ± unconditional 95% confidence interval (CI)] of body condition index (A), haematocrit (B), baseline corticosterone (C) and acute corticosterone (D). Pale blue bars indicate the full data set where renests were categorized according to date; dark blue bars indicate a subset of data with known nest attempts.

**Figure 2:**
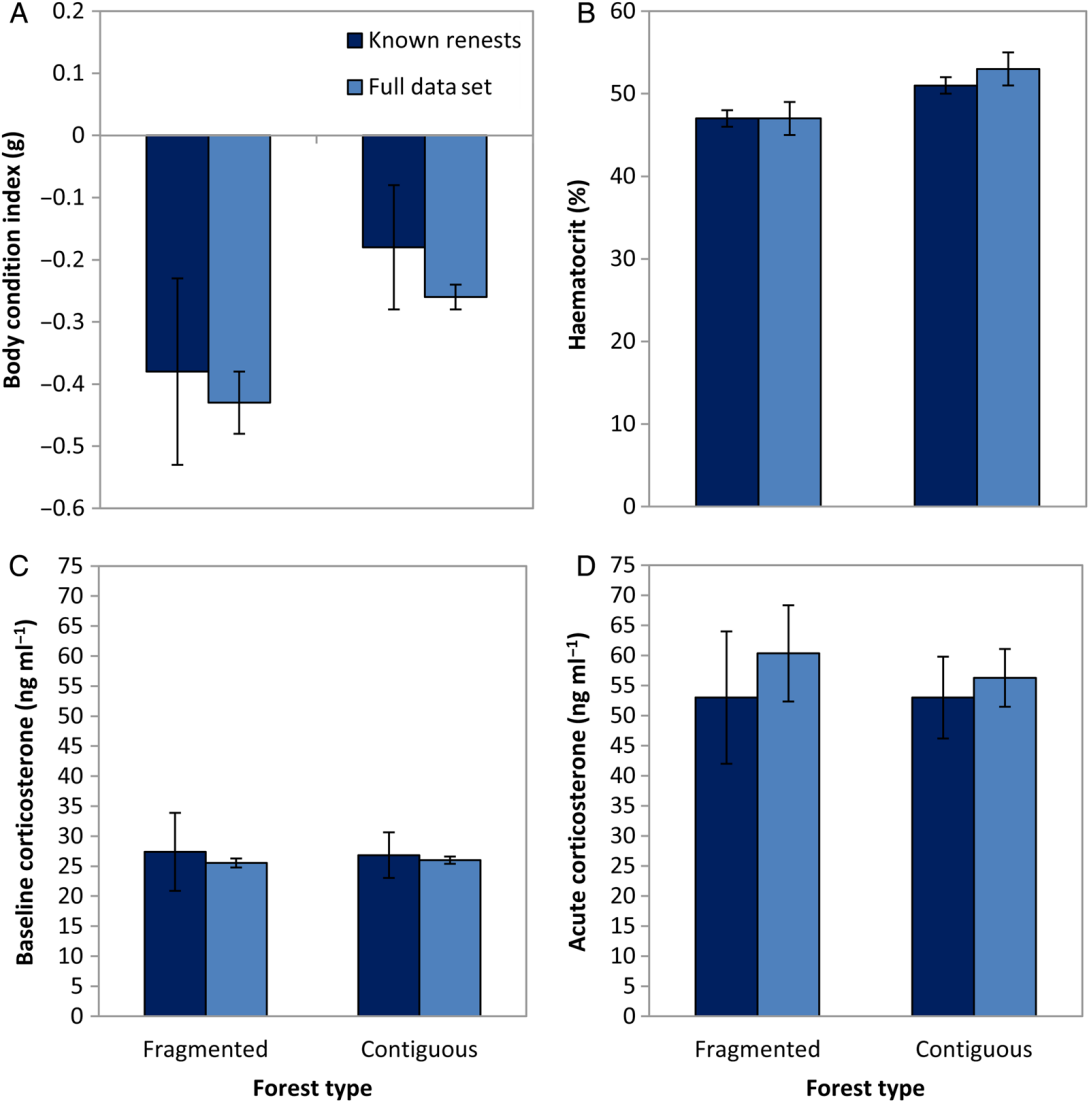
Physiological measures of female indigo buntings with forest type. Parameter estimates (β ± unconditional 95% CI) of body condition index (A), haematocrit (B), baseline corticosterone (C) and acute corticosterone (D) of female indigo buntings in fragmented and contiguous forest. Pale blue bars indicate the full data set where renests were categorized according to date; dark blue bars indicate a subset of data with known nest attempts.

**Figure 3:**
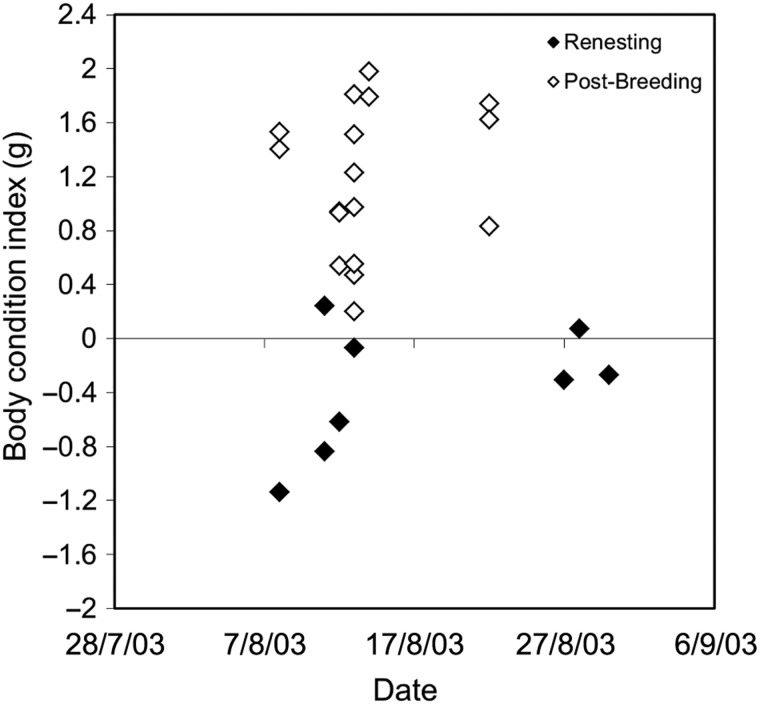
Comparison of renesting and post-breeding females. *Post hoc* analysis showing body condition index of renesting females (*n* = 8) and post-breeding females (*n* = 18) caught at the same time in mid-August in the fragmented forest.

For the full data set and the subset with known attempts, the best model explaining variation in maternal body condition was nesting attempt (relative importance, 0.9 and 0.58, respectively; Table 2). Females that renested one or more times after nest predation had lower body condition than females on their first attempt (Fig. 1A). Forest type had some support, with relative importance of 0.25 and 0.43, and model-averaged estimates showed lower condition in fragments than in contiguous forest (Fig. 2A). We further examined the possible influence of a simple seasonal decline in maternal condition by investigating a subset of females measured during a 3 week period in mid-August. These females were either still provisioning nestlings or had finished breeding and were congregating in food plots to feed. Provisioning females had lower body condition than post-breeding females caught at the same time (Fig. 3).

**Table 2:** Candidate models and model selection results explaining variation in maternal body condition index using a full data set where nesting attempts were categorized as first or renest and a subset of data with known nesting attempts

	Full data set				Subset with known renests		
Model	Body condition index (*n* = 186)			Model	Body condition index (*n* = 79)		
	*k*	ΔAIC_c_	*ω*		*k*	ΔAIC_c_	*ω*
A + Day	4	0.0	0.74	A + Day	4	0	0.53
F + A + Day	6	3.0	0.16	F + Day	4	0.70	0.38
F + Day	4	4.2	0.09	F + A + Day	6	4.62	0.05
Null	1	8.7	0.01	Null	1	5.41	0.04
F + A + F × A + Day	10	11.5	0.00	F + A + F × A + Day	10	14.59	0.00

Model parameters include attempt (A), forest (F) and nuisance variable, day of year.

For the relationship between maternal haematocrit and nesting attempt and forest type, the most supported model for both data sets included forest type (Table 3). Nesting attempt had some support, with relative importance of 0.35 (full data) and 0.29 (known renests). Haematocrit was lower for renesting females (Fig. 1B) and lower in fragmented forest than in contiguous forest (Fig. 2B).

**Table 3:** Candidate models and model selection results explaining variation in maternal haematocrit using a full data set where nesting attempts were categorized as first or renest and a subset of data with known nesting attempts

	Full data set				Subset with known renests		
Model	Haematocrit (*n* = 147)			Model	Haematocrit (*n* = 62)		
	*k*	ΔAIC_c_	*ω*		*k*	ΔAIC_c_	*ω*
F + Day	4	0.0	0.65	F + Day	4	0.0	0.60
F + A + M + Day	7	2.5	0.18	A + Day	4	2.4	0.18
F + A + Day	6	2.8	0.17	F + A + Day	6	3.3	0.11
F + A + F × A + M + Day	11	11.7	0.00	M + Day	3	5.4	0.04
M + Day	3	19.5	0.00	Null	1	5.7	0.03
A + Day	4	19.9	0.00	F + A + M + Day	7	5.7	0.03
Null	1	20.4	0.00	F + A + F × A + M + Day	11	16.9	0.00

Model parameters include attempt (A), forest (F), maternal body condition index (M) and nuisance variable, day of year.

Maternal condition was the highest ranked model in both data sets (relative importance, 0.56 and 0.62; Table 4); explaining variation in maternal baseline corticosterone and nesting attempt had some support (relative importance, 0.43 and 0.40). Baseline corticosterone was negatively related to maternal condition, although effect sizes were small (full set, β = −0.03; 95% CI, −0.08 to 0.03 g; and known renests, β = −0.06; 95% CI, −0.14 to 0.01 g). Baseline corticosterone was higher for renesting females than for females on first attempts (Fig. 1C) but did not differ between forest types (Fig. 2C).

**Table 4:** Candidate models and model selection results explaining variation in maternal baseline corticosterone using a full data set where nesting attempts were categorized as first or renest and a subset of data with known nesting attempts

	Full data set				Subset with known renests		
Model	Baseline corticosterone (*n* = 153)			Model	Baseline corticosterone (*n* = 68)		
	*k*	ΔAIC_c_	*ω*		*k*	ΔAIC_c_	*ω*
M + Time1	3	0.0	0.44	M + Time1	3	0.0	0.51
A + Time1	4	1.0	0.27	A + Time1	4	1.4	0.26
F + Time1	4	2.4	0.13	F + Time1	4	3.1	0.10
A + M + Time1	5	3.0	0.10	A + M + Time1	5	3.5	0.09
F + A + Time1	6	5.0	0.04	F + A + Time1	6	5.6	0.03
A + M + A × M + Time1	7	7.0	0.01	A + M + A × M + Time1	7	7.7	0.01
F + A + M + Time1	7	7.2	0.01	F + A + M + Time1	7	8.3	0.01
F + A + F × A + M + Time1	13	20.4	0.00	F + A + F × A + M + Time1	13	24.3	0.00
Null	1	25.2	0.00	Null	1	24.1	0.00

Model parameters include attempt (A), forest (F), maternal body condition index (M) and nuisance variable, Time1 (time between capture and first blood sample).

Maternal condition was also the highest ranked model in both data sets (relative importance, 0.97 and 0.76); explaining variation in acute corticosterone and nesting attempt had some support (relative importance, 0.27 and 0.40; Table 5). Acute corticosterone was negatively related to maternal condition (full set, β = −0.14; 95% CI, −0.24 to −0.04 g; and known renests, β = −0.08; 95% CI, −0.17 to 0.0001 g). Acute corticosterone was higher for renesting females than for females on first attempts (Fig. 1D) but did not differ between forest types (Fig. 2D).

**Table 5:** Candidate models and model selection results explaining variation in maternal acute corticosterone using a full data set where nesting attempts were categorized as first or renest and a subset of data with known nesting attempts

	Full data set				Subset with known renests		
Model	Acute corticosterone (*n* = 145)			Model	Acute corticosterone (*n* = 62)		
	*k*	ΔAIC_c_	*ω*		*k*	ΔAIC_c_	*ω*
M + Time2	3	0.0	0.72	M + Time2	3	0.0	0.64
A + M + A × M + Time2	7	3.4	0.13	A + Time2	4	3.1	0.14
A + M + Time2	5	4.0	0.10	F + Time2	4	4.2	0.08
A + Time2	4	7.2	0.02	A + M + Time2	5	4.3	0.07
F + A + M + Time2	7	7.3	0.02	Null	1	5.8	0.03
F + Time2	4	8.9	0.01	A + M + A × M + Time2	7	7.2	0.02
F + A + Time2	6	10.8	0.00	F + A + Time2	6	7.4	0.02
Null	1	11.5	0.00	F + A + M + Time2	7	8.7	0.01
F + A + F × A + M + Time2	13	15.8	0.00	F + A + F × A + M + Time2	13	23.2	0.00

Model parameters include attempt (A), forest (F), maternal body condition index (M) and nuisance variable, Time2 (time between capture and second blood sample).

### Clutch size and nestling condition

**Figure 4:**
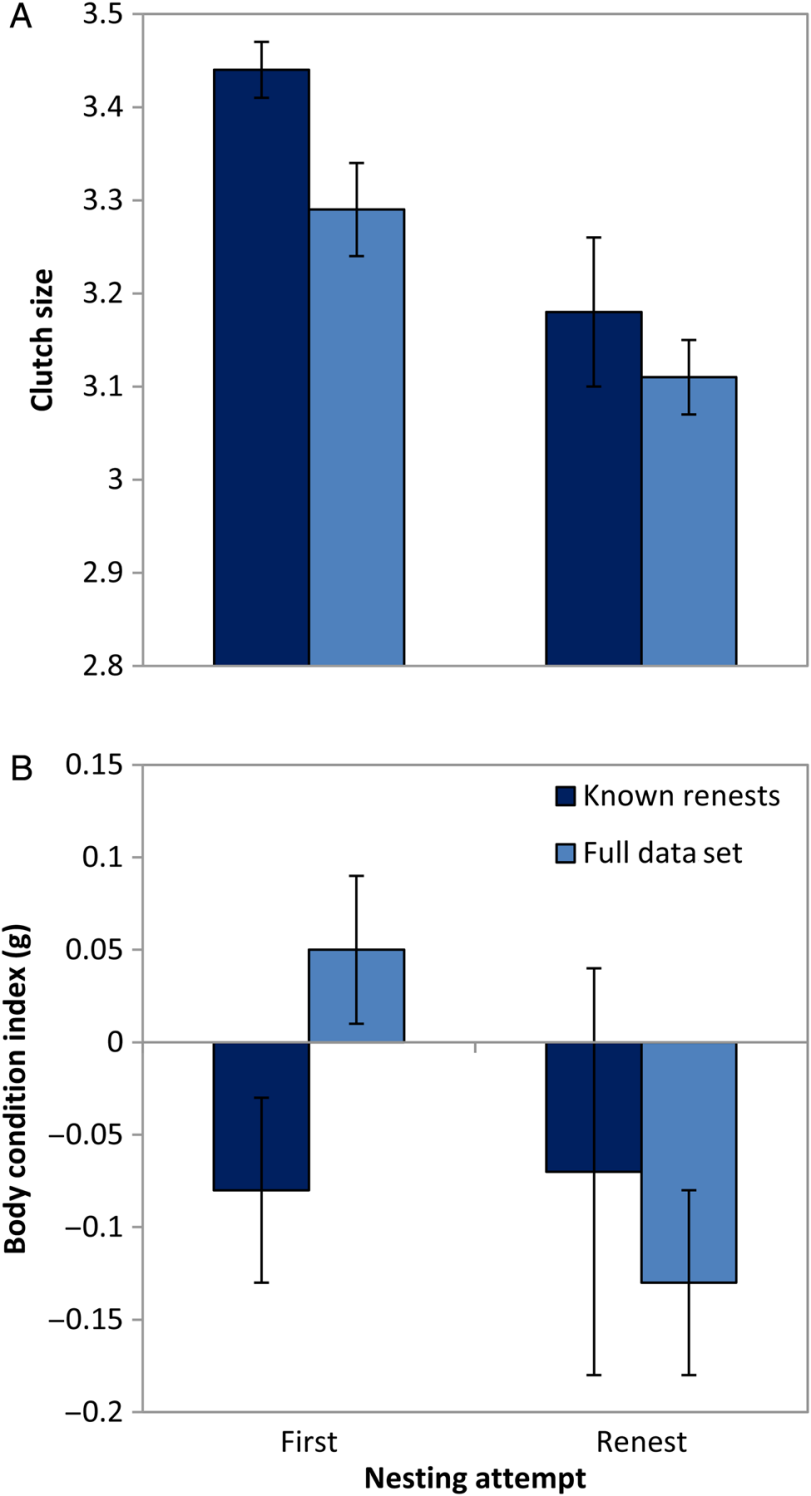
Productivity measures of indigo buntings with nesting attempt. Parameter estimates (β ± unconditional 95% CI) of clutch size (A) and nestling condition (B) of indigo buntings with nesting attempt. Pale blue bars indicate the full data set where renests were categorized according to date; dark blue bars indicate a subset of data with known nest attempts.

**Figure 5:**
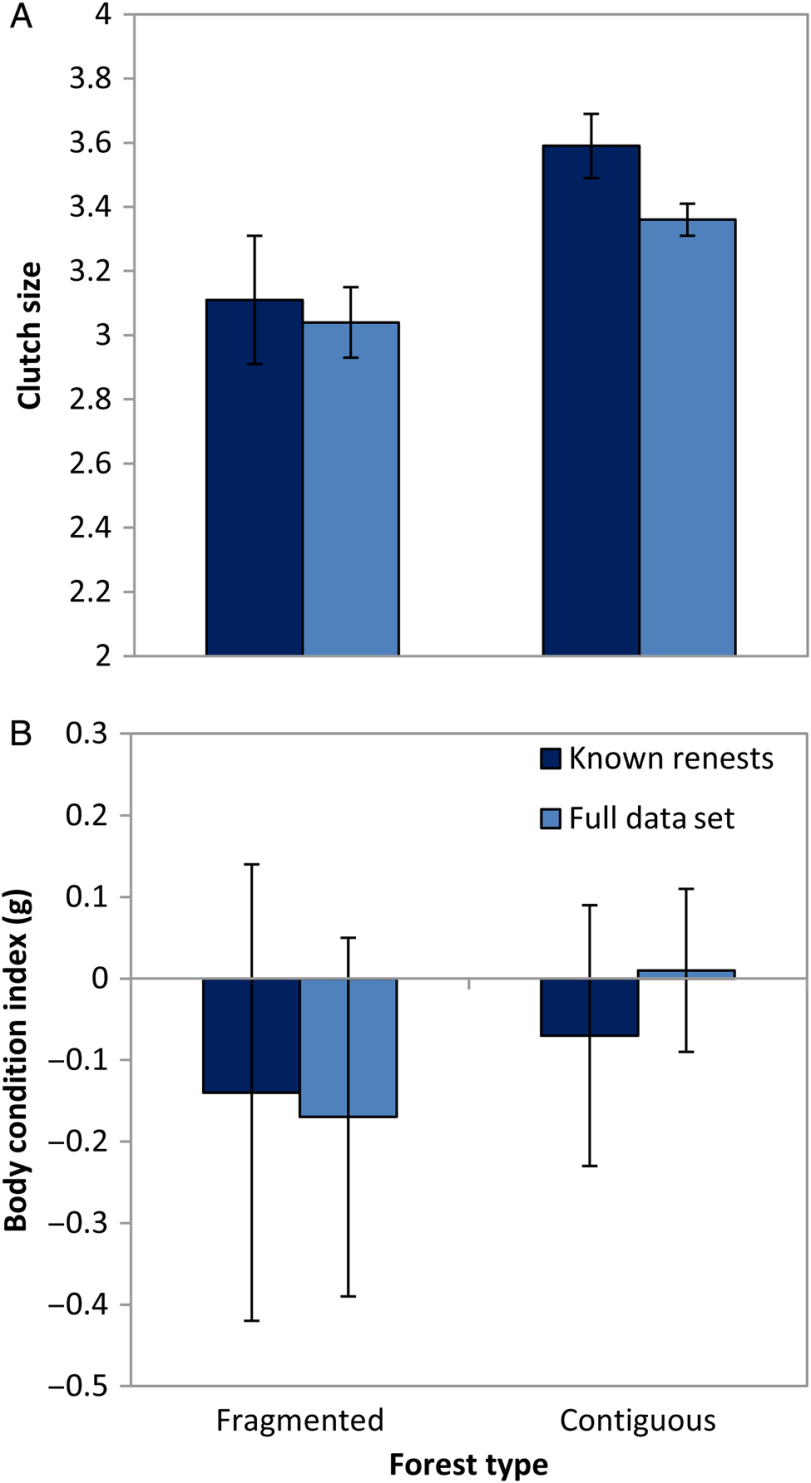
Productivity measures of indigo buntings with forest type. Parameter estimates (β ± unconditional 95% CI) of clutch size (A) and nestling condition (B) of indigo buntings in fragmented and contiguous forest. Pale blue bars indicate the full data set where renests were categorized according to date; dark blue bars indicate a subset of data with known nest attempts.

There was considerable uncertainty in models explaining variation in clutch size. Maternal condition, forest type and nesting attempt each had some support (Table 6). Clutch size was positively related to maternal condition (full set, β = 0.12; 95% CI, 0.01–0.23; and known renests, β = 0.12; 95% CI, −0.02 to 0.26). Model-averaged estimates show that clutch size was lower in renests (Fig. 4A) and in fragmented forests (Fig. 5A). For models related to nestling condition, maternal condition was highest ranked (Table 6), but attempt (relative importance, 0.25 and 0.21) and forest type (relative importance, 0.07 and 0.15) had some support. Nestling condition was positively related to maternal condition (full set, β = 0.17; 95% CI, 0.05–0.28; and known renests, β = 0.07; 95% CI, −0.02 to 0.16). Model-averaged estimates show that nestling condition was lower in renests in the full data set (Fig. 4B) but showed no difference between forest types (Fig. 5B).

**Table 6:** Candidate models and model selection results explaining variation in clutch size and nestling body condition index where nesting attempts were categorized as first or renest and a subset of data with known nesting attempts

	Full data set				Subset with known renests		
Model	Clutch size (*n* = 161)			Model	Clutch size (*n* = 69)		
	*k*	ΔAIC_c_	*ω*		*k*	ΔAIC_c_	*ω*
M + Day	3	0.0	0.29	F + Day	4	0.0	0.44
F + A + M + Day	7	0.3	0.25	F + A + M + Day	7	1.6	0.20
F + Day	4	0.4	0.24	M + Day	3	1.7	0.18
F + A + Day	6	1.2	0.16	F + A + Day	6	2.3	0.14
A + M + Day	5	3.9	0.04	A + M + A × M + Day	5	6.3	0.02
A + Day	4	6.1	0.01	A + Day	4	6.6	0.02
A + M + A × M + Day	7	8.2	0.00	F + A + F × A + M + Day	13	10.6	0.00
F + A + F × A + M + Day	13	8.5	0.00	A + M + Day	7	10.7	0.00
Null	1	53.8	0.00	Null	1	22.7	0.00
	Nestling body condition (*n* = 458)				Nestling body condition (*n* = 216)		
M + Day	3	0.0	0.72	M + Day	3	0.0	0.58
A + M + Day	5	2.9	0.17	F + Day	4	2.6	0.15
F + A + M + Day	7	4.9	0.06	A + Day	4	2.9	0.14
A + M + A × M + Day	7	6.9	0.02	A + M + Day	5	4.2	0.07
A + Day	4	8.1	0.01	Null	2	6.0	0.03
F + Day	4	9.2	0.01	F + A + Day	6	6.9	0.02
F + A + Day	6	9.5	0.00	F + A + M + Day	7	8.2	0.01
Null	2	10.5	0.00	A + M + A × M + Day	7	8.4	0.01
F + A + F × A + M + Day	13	15.2	0.00	F + A + F × A + M + Day	13	21.1	0.00

Model parameters include attempt (A), forest (F), maternal body condition index (M) and nuisance variable, day of year.

### Seasonal differences in condition

Model selection results reveal that the global model was supported in explaining variation in female body condition from pre-breeding to the post-breeding period (Table 7). Before breeding, there was no difference in maternal body condition between forest types (fragmented, β = 0.48; 95% CI, −0.07 to 1.03 g; and contiguous, β = 0.59; 95% CI, 0.04–1.14 g). During breeding, maternal condition was lower in the fragmented forests (see Fig. 1A). During the post-breeding period, there was no difference in maternal body condition between forest types (fragmented, β = 0.99; 95% CI, 0.70–1.28 g; and contiguous, β = 0.49; 95% CI, 0.17–0.80 g). During the post-breeding period, the body condition of juvenile buntings did not differ between forest types (fragmented, β = −0.06; 95% CI, −0.26 to 0.14 g; and contiguous, β = 0.02; 95% CI, −0.16 to 0.20 g).

**Table 7:** Models selection results comparing female body condition from pre-breeding to the post-breeding period (*n* = 266) and juvenile condition (*n* = 116) during the post-breeding period

Response variable	Model	*k*	ΔAIC_c_	*ω*
Female condition	Forest, period, forest × period	12	0.00	1.00
	Null	1	61.04	0.00
Juvenile condition	Null	1	0.00	0.88
	Forest, period, forest × period	3	3.83	0.12

## Discussion

Our results provide evidence that predator-induced renesting negatively affects maternal body condition and demonstrate how renesting could still result in lower reproductive gains compared with females that are successful on their first attempt. There was no difference in maternal body condition between the two forest types prior to nest initiation, indicating that both areas were settled by females of similar quality. Nest survival was lower in the fragmented forest, causing a greater proportion of females to renest. Poor-conditioned females could contribute to an increased risk of predation resulting from changes in parental activity or increased nestling begging (Martin *et al*., 2000; Haskell, 2002), but maternal body condition index was not an important predictor of nest survival in our study. Renesting females had lower body condition and haematocrit and higher baseline and acute corticosterone than females on their first attempt. Our results demonstrate that declines in maternal condition with renesting were not the result of a simple seasonal decline but were more likely to be associated with proximate energetic demands.

Further anecdotal evidence showed that renesting females provisioning nestlings were in poorer condition than post-breeding females caught during the same 1 week period in mid-August in the forest fragments. Perhaps females were unable to regain lost body mass before incubating replacement clutches within 3–5 days after nest failure. Once released from provisioning, females might regain condition rather quickly, but when forced into a renesting cycle by nest predation, the opportunity to regain condition is limited.

Several studies have found that experimentally increasing egg production lowers maternal body condition and haematological values (Kalmbach *et al*., 2004; Wagner *et al*., 2008; Travers *et al*., 2010), hatching success (Kalmbach *et al*., 2004) and interseasonal survival (Nager *et al*., 2001; Visser and Lessells, 2001; although see Hoover, 2003). In our study, lower haematocrit in renesting females indicates increased egg production and physiological demand.

The difference in haematocrit between forest types could be due to differences in habitat quality or resource-based limitations. However, others have found that the change in haematocrit of laying females is independent of the quality of the diet (Wagner *et al*., 2008). Williams (2005) and Wagner *et al*. (2008) suggest that the physiological constraints of egg production could be linked to non-resource-based mechanisms, such as oxidative stress (Monaghan *et al*., 2009) or pleiotropic effects of maternal hormones on other physiological systems (Ketterson and Nolan, 1999). Provisioning females in our fragmented sites had lower body condition than post-breeding females caught during the same 3 week period, suggesting that non-resource-based mechanisms were influencing maternal performance (Williams, 2005) rather than resource-based limitations.

Lean individuals might be expected to have a more robust response to stressors because they have lower energy reserves to compensate for the stressor (Wingfield *et al*., 1995; Romero, 2002). Higher baseline corticosterone but lower body condition in renesting buntings indicates that the effects of corticosterone were not sufficient to stimulate energy intake and maintain body mass (Astheimer *et al*., 1992; Landys *et al*., 2006). The negative relationship between maternal body condition and acute (stress-induced) corticosterone concentrations found in our study is consistent with previous studies on breeding passerines (Hau *et al*., 2010), seabirds (Kitaysky *et al*., 1999; Heidinger *et al*., 2006) and waterfowl (Perfito *et al*., 2002). Predator-induced stress has been shown to elevate corticosterone of parents (Scheuerlein *et al*., 2001; Clinchy *et al*., 2011), and perceived predation risk has been linked to reduced productivity (Saino *et al*., 2005; Travers *et al*., 2010; Zanette *et al*., 2011). Maternal corticosterone can be passed to eggs (Hayward and Wingfield, 2004; Saino *et al*., 2005), and experimentally stressed females have been reported to produce clutches with lower hatchability and lower offspring quality (Hayward and Wingfield, 2004; Travers *et al*., 2010). Recent studies suggest that exposure to predators or even predator cues can have sustained effects that may be linked to demographic processes (Lima, 2009; Clinchy *et al*., 2011).

As predicted, renesting and reduced maternal condition had negative implications for productivity in our study. Nestling condition and clutch size were positively related to maternal condition. Nestling condition and clutch size were lower in renest attempts than in first attempts, indicating indirect predator effects reported by experimental studies (Travers *et al*., 2010; Zanette *et al*., 2011). Likewise, clutch size was lower in fragmented forests, where a higher proportion of nests failed on the first attempt. Nestling condition tended to be lower in fragmented forests, but this difference was evident in the full data set only. When nest predation is high, Slagsvold (1984) suggests that smaller broods are adaptive, in that energy is conserved per attempt when repeated attempts are necessary during a single breeding season. Thus, if females adjust clutch sizes with predation pressure to maximize their own condition and that of their offspring (Smith and Fretwell, 1974; Nur, 1986), and because there is a greater physiological cost associated with incubating larger clutches (DuRant *et al*., 2013), then smaller clutches might be expected to contain fewer but high-quality young and could explain the lack of difference in nestling condition between forest types. Further study is needed to disentangle the effects of physiological constraints and behavioural strategies on clutch size adjustments and allocation of resources to offspring. However, rather than viewing maternal effects mainly as energy trade-offs (Slagsvold, 1984; Martin, 1995), future work should focus on whether resource-based limitations interact with physiological mechanisms or behavioural strategies that result in long-term implications for females and offspring (Love and Williams, 2008; Monaghan *et al*., 2009).

We predicted that post-breeding females and juveniles in the fragmented forests would be in poorer condition, given their greater reproductive effort and prolonged breeding period. In fact, we observed six renesting females in the fragments in late August that had been abandoned by their mates shortly after egg-laying. However, despite a breeding season that was 2 weeks longer in the fragmented forests, we found that post-breeding condition tended to be higher, suggesting that food availability was not limiting in those sites. Although environmental conditions in our fragmented sites seemed less favourable, post-breeding foraging opportunities might have allowed adult females and juveniles to recover body condition quickly and compensate for a prolonged breeding season. The extensive matrix of early successional habitats in some fragmented forests enhances foraging opportunities (Rodewald and Brittingham, 2004; Vitz and Rodewald, 2006) and protection from non-nest predators (Anders *et al*., 1998). Although the post-breeding condition of females and juveniles was not lower in fragmented sites, the possibility exists for carry-over effects on subsequent life-history stages, given that juvenile survival is lower for young fledged from late broods (Verboven and Visser, 1998; Drent, 2006). Several studies show that late-fledged young have lower annual survival, presumably because they are in poorer condition and have less experience and preparation prior to migration (Nilsson and Svensson, 1996; Tarof *et al*., 2011; McKim-Louder *et al*. 2013).

### Conclusions

Our findings are consistent with other studies that show lower productivity in forest fragments (Porneluzi and Faaborg, 1999; Burke and Nol, 2000). Our findings are rare, in that they provide evidence that predator-induced renesting negatively affects maternal condition and demonstrate how renesting could still result in lower reproductive gains via smaller clutch sizes and lower offspring quality. Our study is unique in extending observations to the post-breeding period, where we found that renesting prolongs the breeding season, but juveniles produced in the fragments did not exhibit lower body condition. Given the potential for body condition to rebound during the post-breeding period, further work should investigate how predator-induced costs during breeding could be mediated during the post-breeding period by the degree and context of fragmentation.

Whether renesting and prolonged breeding have consequences for lifetime reproductive success is unknown, but effects could be especially important in species with strong patterns of migratory connectivity (Marra *et al*., 2006; Norris and Marra, 2007). Delays in migration or arrival at wintering sites in poor condition might reduce access to high-quality wintering habitat (Marra and Holberton, 1998), delay arrival to breeding sites the following year (Marra *et al*., 1998; Norris *et al*., 2004) and lower subsequent reproductive performance (Reudink *et al*., 2009). Furthermore, if birds respond to increased predation risk by limiting clutch size or the number of offspring produced (Suarez *et al*., 1997; Travers *et al*., 2010; Zanette *et al*., 2011), due to behavioural strategies or physiological constraints, then nest predation has additional demographic consequences for populations that should be investigated in the context of habitat studies. The effects of renesting on productivity and energetics demonstrated here, along with the potential for carry-over effects, suggests that further work is needed to understand how individual responses to indirect predator effects could scale up to demographic consequences.

## Supplementary material

Supplementary material is available at *Conservation Physiology* online.

## Funding

This work was supported by the National Center for Environmental Research (NCER) STAR Program, Environmental Protection Agency (grant number U916163), University of Missouri Conservation Biology Program Fellowship and the US Forest Service North Central Research Center.

## Supplementary Material

Supplementary Data
