# Supplementary material for: Predator-induced renesting and reproductive effort in indigo buntings: more work for less pay?
Source: Conserv Physiol. 2015 Feb 5;3(1):cou063. doi: 10.1093/conphys/cou063 (PMC4778466; doi:10.1093/conphys/cou063)
Supplement: Supplementary Data [file cou063morrissuppl.docx]

**Supplementary Information for**

Predator-induced renesting and maternal effort in Indigo Buntings: more work for less pay?

Authors

Dana L. Morris,^*^ John Faaborg, Brian E. Washburn, and Joshua J. Millspaugh

^*^Corresponding author: [dlmorris@centralmethodist.edu](mailto:dlmorris@centralmethodist.edu)

**This file includes:**

Tables S1 to S3

Table S1. Model selection results of nuisance parameters

explaining variation in nest survival. Parameters with

ΔAIC_c_ < 4 were included in final candidate models.

| **Model** | **k** | **ΔAIC_c_** | **ω** |
| --- | --- | --- | --- |

| **Nuisance set (n=1784)** |  |  |  |
| --- | --- | --- | --- |
| Stage | 2 | 0.0 | 0.60 |
| Day | 2 | 1.1 | 0.34 |
| Habitat | 3 | 4.5 | 0.06 |
|  |  |  |  |
| **Maternal body condition (n=759)** |  |  |  |
| Null | 1 | 0.0 | 0.71 |
| Maternal condition^a^ | 2 | 1.8 | 0.29 |

^a^ Effect size of maternal condition was negligible

(β = -0.07; 95% CI: -0.33 – 0.19) and was not included in final

candidate models.

Table S2. Model selection results of nuisance parameters

explaining variation in maternal measures of condition.

Results were obtained using the full data set. Parameters

with ΔAIC_c_ < 4 were included in final candidate models.

| **Model** | **k** | **ΔAIC_c_** | **ω** |
| --- | --- | --- | --- |
|  |  |  |  |
| **Body condition index** |  |  |  |
| Day | 2 | 0.0 | 0.81 |
| Brood | 2 | 4.1 | 0.10 |
| Habitat | 5 | 5.2 | 0.06 |
| Nestlage | 3 | 7.4 | 0.02 |
| FemAge | 3 | 8.9 | 0.01 |
| Bhco | 3 | 9.4 | 0.01 |
|  |  |  |  |
| **Hematocrit** |  |  |  |
| Day | 2 | 0.0 | 0.93 |
| Brood | 2 | 6.3 | 0.04 |
| Nestlage | 2 | 8.6 | 0.01 |
| Habitat | 5 | 10.1 | 0.01 |
| Bhco | 3 | 10.2 | 0.01 |
| FemAge | 3 | 11.1 | 0.00 |
|  |  |  |  |
| **Baseline corticosterone** |  |  |  |
| Time1 | 2 | 0.0 | 1.00 |
| Day | 2 | 25.4 | 0.00 |
| Brood | 2 | 25.7 | 0.00 |
| Nestlage | 2 | 26.3 | 0.00 |
| FemAge | 3 | 28.8 | 0.00 |
| BHCO | 3 | 28.9 | 0.00 |
| Habitat | 5 | 32.3 | 0.00 |
|  |  |  |  |
| **Acute corticosterone** |  |  |  |
| Time2 | 2 | 0.0 | 0.90 |
| Nestlage | 2 | 5.2 | 0.07 |
| Day | 2 | 8.2 | 0.01 |
| Brood | 2 | 8.5 | 0.01 |
| Age | 3 | 10.1 | 0.01 |
| BHCO | 3 | 10.4 | 0.00 |
| Habitat | 5 | 13.6 | 0.00 |

Table S3. Model selection results of nuisance parameters

explaining variation in clutch size and nestling body condition.

Results were obtained using the full data set. Parameters with

ΔAIC_c_ < 4 were included in final candidate models.

| **Model** | **k** | **ΔAIC_c_** | **ω** |
| --- | --- | --- | --- |

| **Clutch size** |  |  |  |
| --- | --- | --- | --- |
| Day | 2 | 0.0 | 1.00 |
| Habitat | 5 | 44.9 | 0.00 |
| FemAge | 3 | 45.4 | 0.00 |
|  |  |  |  |
| **Nestling body condition** |  |  |  |
| Day | 2 | 0.0 | 0.70 |
| Nestlage | 2 | 4.0 | 0.09 |
| Brood | 2 | 4.3 | 0.08 |
| FemAge | 3 | 4.7 | 0.07 |
| Bhco | 3 | 5.6 | 0.04 |
| Habitat | 5 | 6.9 | 0.02 |
